# Stereotypes About Enterotype: the Old and New Ideas

**DOI:** 10.1016/j.gpb.2018.02.004

**Published:** 2019-04-23

**Authors:** Mingyue Cheng, Kang Ning

**Affiliations:** Key Laboratory of Molecular Biophysics of the Ministry of Education, Hubei Key Laboratory of Bioinformatics and Molecular-imaging, Department of Bioinformatics and Systems Biology, College of Life Science and Technology, Huazhong University of Science and Technology, Wuhan 430074, China

**Keywords:** Enterotype, Gut microbiome, Biomarker, Continuity, Computational methods

## Abstract

In 2011, the term “**enterotype**” first appeared to the general public in *Nature*, which refers to stratification of human gut microbiota. However, with more studies on enterotypes conducted nowadays, doubts about the existence and robustness of enterotypes have also emerged. Here we reviewed current opinions about enterotypes from both conceptual and analytical points of view. We firstly illustrated the definition of the enterotype and various factors influencing enterotypes, such as diet, administration of antibiotics, and age. Then we summarized lines of evidence that pose the concept against the enterotype, and described the current methods for enterotype analysis. Finally, we showed that the concept of enterotype has been extended to other ecological niches. Based on current studies on enterotypes, it has been clear that more studies with larger sample sizes are needed to characterize the enterotypes. Improved **computational methods** are also required to build sophisticated models, reflecting the dynamics and resilience of enterotypes.

## The definition of enterotypes

Each individual is different not only due to his/her own genetic materials but also the gut microbiome. The human gut microbiome consists of at least 1800 genera and approximately 15,000–36,000 species of bacteria [1], with the total number of bacterial cells ranging from 10^13^ to 10^14^, which is of the same order as the number of human cells (3.0 × 10^13^) [2]. Gut microbiome also contains more than100 times more genes, compared with 25,000 genes in humans [3]. As for the functions, gut microbiome has played a vital role in human body. For example, they can degrade a variety of otherwise indigestible dietary polysaccharides and synthesize essential amino acids and vitamins [4]. Furthermore, the dysbiotic microbiota can lead to the loss of regulatory immune effects on the gut mucosa, which is associated with a number of inflammatory and immune-mediated diseases [5], [6].

Gut microbiota vary largely among individuals in time and space scale [7], which has been regarded as an obstacle to the gut microbiome-based medical applications. The enterotype concept raised in 2011 might make this obstacle possible to be coped with. Arumugam et al. [8] analyzed 33 qualified samples from different populations and found that these samples can be stratified into three distinct robust clusters driven by discriminative genera. These include *Bacteroides* (enterotype 1), *Prevotella* (enterotype 2), and *Ruminococcus* (enterotype 3), with each cluster designated as an enterotype subsequently [8]. The essence of enterotyping is to stratify human gut microbiome, serving as a process of dimensionality reduction to collapse global microbiome variation into a few categories. These categories, named “enterotypes”, were reported originally as “*densely populated areas in a multidimensional space of community composition*,” which means that they are not sharply delimited as human blood types [8].

Following the concept of enterotype, subsequent studies have demonstrated that enterotypes are quite robust among populations. For instance, Arumugam et al. [8] apply enterotype analysis to two large published gut microbiome datasets (85 metagenomes of Danish individuals from a published Illumina dataset and 154 pyrosequencing-based 16S sequences from American individuals) and have detected three enterotypes. Moreover, Liang et al. [9] have observed three enterotypes when investigating 181 human fecal samples from adults in Taiwan, China. Besides the two enterotypes identified by *Bacteroides* and *Prevotella* as described before, a third enterotype is identified by family Enterobacteriaceae, suggesting that there might be a new enterotype in the Asian population. Nevertheless, whether it can be regarded as a feature of Asian microbiome or it is produced by chance needs further investigations.

However, with more studies focusing on the stratification of human gut microbiome, it has been noticed that the number of enterotypes varies when different methods are employed even on the same samples [9], [10], [11]. For example, Wu et al. [11] used different distance matrices including weighted/unweighted UniFrac, Euclidean, the Bray–Curtis, and the Jensen–Shannon divergence (JSD) to stratify the gut microbiota samples of 98 healthy volunteers and have found that most analyses reveal two enterotypes with stronger support, whereas only the analysis using weighted UniFrac distance clearly shows three enterotypes. Hence, they claimed that *Bacteroides* enterotype is fused with the less well-distinguished *Ruminococcus* enterotype. Liang et al. [9] performed 9 β-diversity matrices on enterotype analysis using three clustering methods and obtained inconsistent numbers of enterotypes based on various evaluation scores. Moreover, several other studies have confirmed that according to the microbiome profiles, samples can only be stratified into two enterotypes represented by *Bacteroides* and *Prevotella*, respectively [12], [13], [14], [15]. Therefore, it remains to be considered whether the *Ruminococcus* enterotype or other so-called “the third enterotypes” should be abandoned and, instead, fused with the *Bacteroides* or/and *Prevotella* enterotypes, due to their less significance among gut microbiome samples [11], [16] and the analytical bias [10], [11].

## Enterotype is influenced by various factors

Since enterotypes are defined based on gut microbiota, which changes rapidly in response to interventions [11], [17], [18], [19], [20], it is conceivable that enterotypes are not constant for individuals, but rather dynamically affected by various factors as well. However, the alternation of microbiota composition in a short term might not be sufficient to switch the enterotype [11], due to the reversibility and relative stability of gut microbiota [21], [22], [23], [24]. Dietary intake and administration of antibiotics are known to significantly impact our gut microbiota [11], [25], [26]. In addition, many factors such as the diet, the life style, and environmental stress vary during different age stages [27], [28], making age a combination of these factors that largely impacts both enterotype patterns and identification.

### The effects of diet

The diet has been reported to affect gut microbiota composition in multiple studies [11], [19], [29], [30], [31], [32], [33], [34]. Although the short-term diet adjustment might not be able to change the enterotype, the long-term diet has been observed to significantly associate with the enterotype patterns [11].

The short-term diet adjustment (usually lasting less than a month) can cause a rapid and significant change in microbiota composition [26], [35], which, however, might not lead to stable switches between enterotypes [11]. One controlled-feeding study involving 10 subjects has shown that the variation of gut microbiota composition is observed in 24 h after intake of high-fat/low-fiber or low fat/high-fiber diet, but enterotypes remain stable during the 10 days of dietary intervention [11]. Only one subject switches from *Bacteroides* enterotype to *Prevotella* enterotype, which then reverts the next day [11]. Exceptions have been found in another 6-week controlled feeding trial, when investigating the effects of dietary capsaicin on gut microbiota. Two subjects of *Prevotella* enterotype switch to *Bacteroides* enterotype during the high-capsaicin period and at the end of washout period, respectively. Nevertheless, their relative abundance of *Prevotella* is still much higher than that in the subjects of *Bacteroides* enterotype. Additionally, enterotypes of the other 10 subjects remain stable during this 6-week controlled feeding trial [15]. These observations indicate that the impact of short-term dietary intervention on gut microbiota is not strong enough to change the enterotype.

During long-term environmental changes, the composition of an individual’s gut microbiota is predominantly determined by dietary habits [11], [36] and such dynamics is highly variable among individuals [37], [38]. Wu et al. [11] claimed that *Bacteroides* enterotype favors protein and animal fat, characterized by meat consumption as in a Western diet, whereas *Prevotella* enterotype prefers carbohydrates and simple sugars, which are typical of the carbohydrate-based diet in agrarian societies [11], [14]. Whether long-term dietary interventions (usually lasting more than several months) can stably change enterotypes still remains unknown. Interestingly, a recent study by Liu et al. [26] has revealed that even after half a year, the enterotypes of the Chinese individuals can be reverted after the subjects shift back to their routine diets. Therefore, the impact of long-term dietary shift needs to be further investigated.

### The effects of antibiotics

Antibiotics have been widely used over the world nowadays, which can have both temporary and permanent effects on our gut microbiota [39], [40] through various mechanisms [41], [42]. In a recent study, by administering cefprozil, a second-generation cephalosporin, to 18 healthy volunteers, Raymond et al. [43] have observed the increased abundance of several bacterial genera such as *Flavonifractor*, *Lachnoclostridium*, and *Parabacteroides*, but a decrease in the abundances of several bacterial families such as Bifidobacteriaceae and Coriobacteriaceae. Six of these 18 exposed participants have their level of *Enterobacter cloacae* complex bacteria increased from an average of 0.0001% to 0.1% after a 7-day course of cefprozil. It is worth noting that five of these six participants initially contain a *Bacteroides* enterotype with lower bacteria diversity [43]. This finding demonstrates that the effect of cefprozil exposure may be linked to a *Bacteroides* enterotype. Therefore, not only the antibiotics themselves can have effects on our gut microbiota, but also these effects are likely influenced by the enterotypes of subjects. Thus, enterotype might be taken into account when considering therapeutic antibiotic intervention.

### Correlation with age

In the original paper about enterotypes [8], age has been described as not associated with enterotypes. This conclusion is obtained based on the observation that the distribution of age seems not significantly different among different enterotypes. However, the stability and composition of human gut microbiota vary largely across different age stages in human life (infancy, childhood, adulthood, and elderhood) [28], [44], [45], [46]. That is to say, the results of enterotype analysis would be different when using gut samples of subjects at different age stages. A recent study has reported three enterotypes of children at school age, driven by *Bacteroides*, *Prevotella*, and *Bifidobacterium*, respectively [47]. They are different from the enterotypes reported in the adults. Moreover, the gut microbiota of infants and the elderly is very dynamic [19], [27], [28], making it unsuitable for analysis of a “stable status”.

Gut microbiota of infants has been reported to be quite unstable [28], [46]. Firstly, their gut microbiota can be influenced by the mode of delivery: vaginally-delivered infants differ from those delivered by caesarean section, both in terms of the timing of colonization and the composition of their microbiota [24], [48], [49], [50]. Additionally, the gut microbiota profile of preterm babies differs from that of full-term babies [50], [51]. In the first year of life, the gut of infants is quite sterile initially and gradually becomes extremely densely colonized with bacteria. Finally, the gut microbiota of infants ends with a profile largely similar to that in adults [28], [52]. This process of evolution of the gut microbiota of infants is still poorly understood. Not to mention that many key events, such as the solid food intake and administration of antibiotics, can have effects on gut microbiota of infants as well [48].

The gut microbiota composition of the elderly (>65 years) varies extremely between individuals [19]. Additionally, the core microbiota of the elderly is quite different from that of young adults, with a greater proportion of *Bacteroides* spp. and distinct abundance patterns of *Clostridium* groups [27].

Gut microbiota profiles are quite distinct among different stages of human life [28], [44], [45], [46]. Along with aging, changes of life-style happen, such as the dietary habits, the frequency and variety of administration of antibiotics, as well as the human activities. As a result, the age can be regarded as a combination of multiple factors affecting human enterotypes. Age can also be a confounding factor when choosing samples for enterotype analysis. It might not be appropriate to mix the unstable microbiota samples of the infants and elderly with those of adults when performing enterotype analysis. Therefore, the investigations should be conducted separately for these age groups.

## The concept against enterotypes

### Enterotype might be continuous

**Figure 1:**
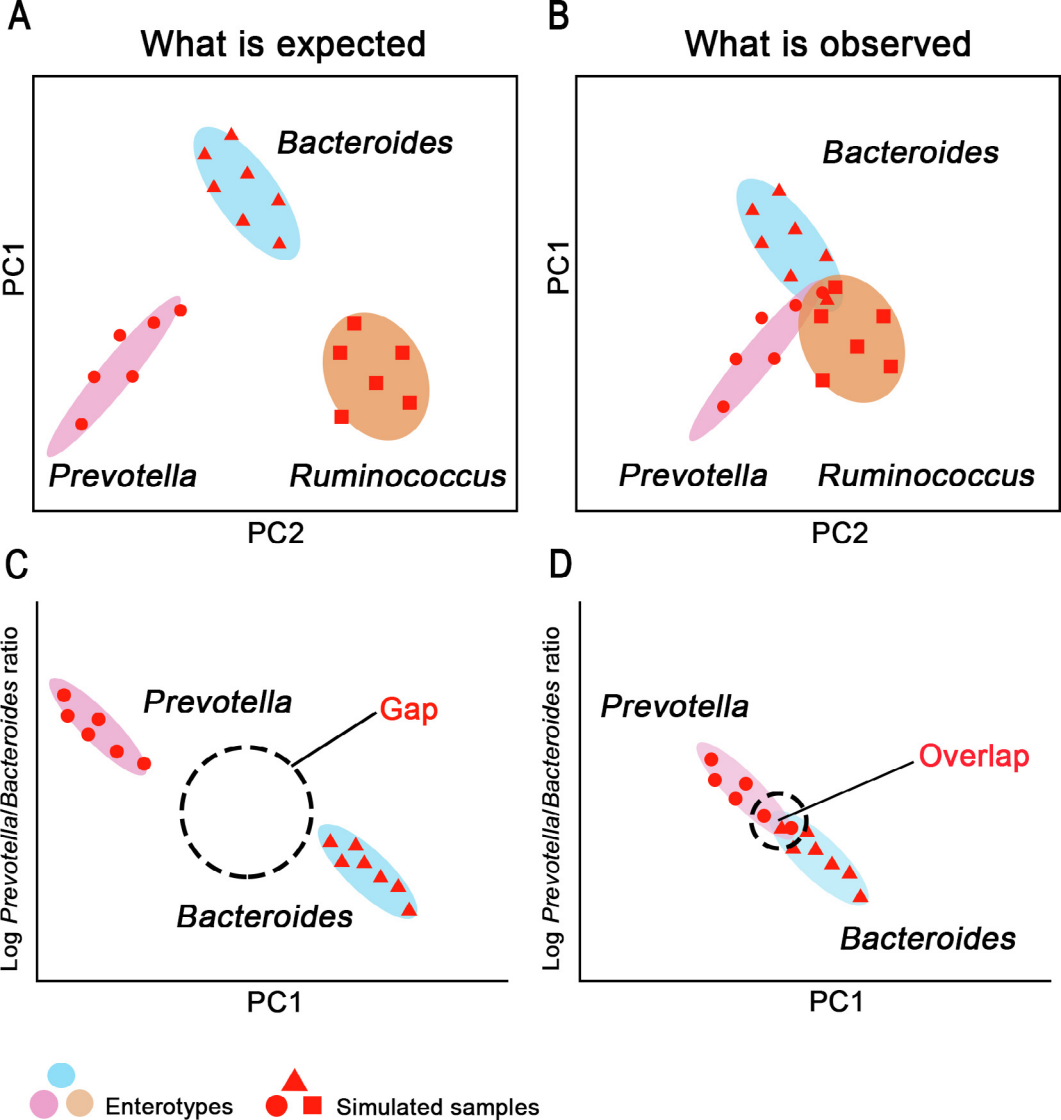
**Enterotypes might be continuous rather than discrete** Simulated microbiota samples are indicated using dots, triangles, or squares with different shapes representing different patterns of microbiota composition that these samples initially contain. The simulated samples are grouped into three enterotypes (*Prevotella*, *Bacteroides*, and *Ruminococcus*) according to their microbiota composition, plotted in the scenario showing what is expected to see (**A**) and what is actually observed (**B**). The log ratio of the relative abundance of *Prevotella* to *Bacteroides* of these simulated samples is plotted against the first principal coordinates in panels A and B, to show the expected scenario (**C**) and the observed scenario (**D**).

In principle, enterotypes can clearly separate samples, and such separation can be clearly observed in principal coordinate analysis (PCoA), as is shown in Figure 1A. However, a number of studies especially with larger sample size [10], [16], [53], [54] have indicated that samples from different enterotypes cannot be separated clearly into distinct clusters (Figure 1B). Moreover, when looking at the gradient of log ratio of *Bacteroides* to *Prevotella*, the discrete enterotypes are expected to show an obvious gap of the gradient, thus stratifying these samples into clusters (Figure 1C). Nevertheless, in most cases, a continuous gradient rather than a gap is observed [11], [55] (Figure 1D). Therefore, doubts about the presence of discrete enterotypes emerge [16], [55]. Instead of discrete enterotypes, this kind of distribution is deemed as a continuum changing from *Bacteroides*-driven to *Prevotella*-driven microbiota types [55].

### Enterotype is not always stable

**Figure 2:**
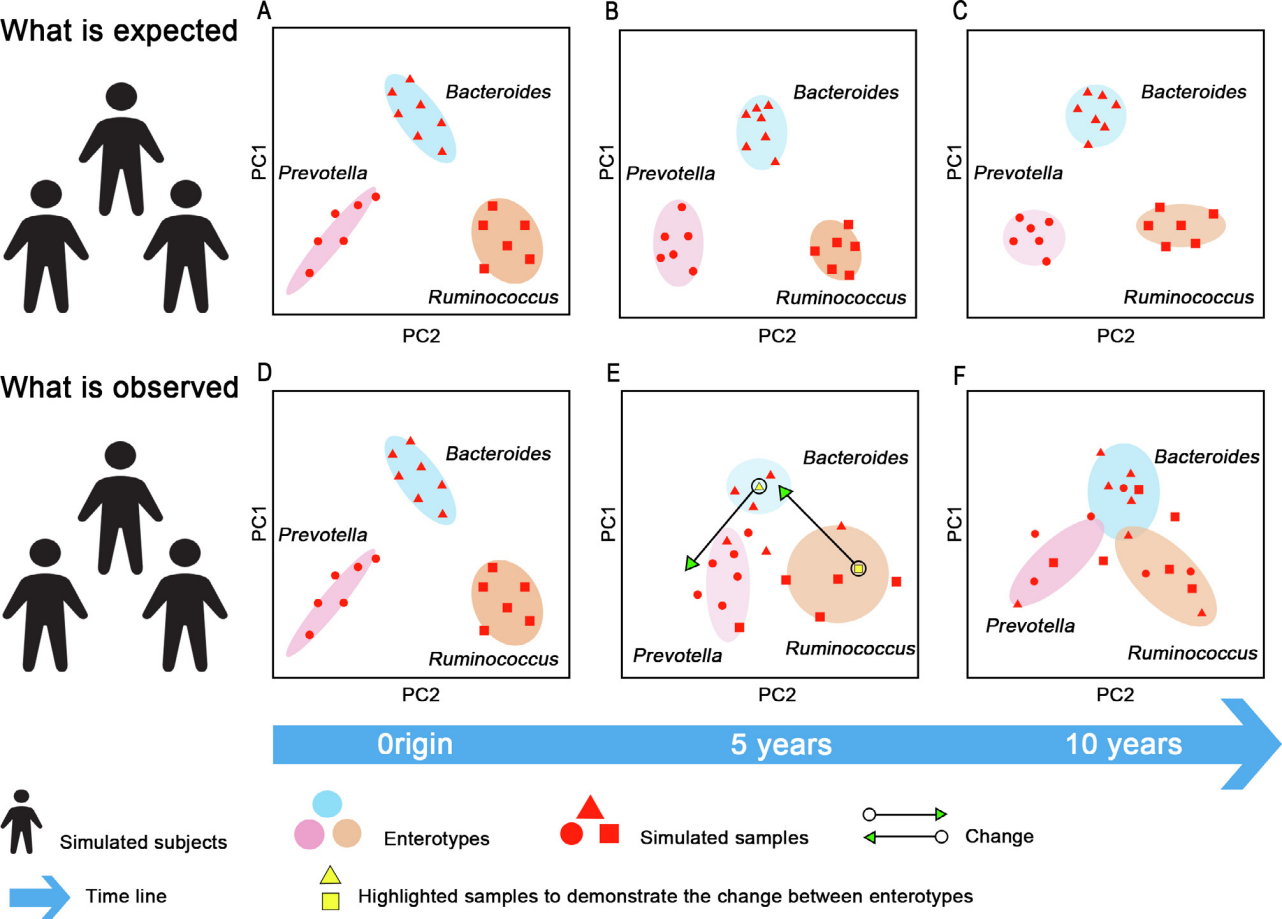
**Enterotype might not always be stable** Simulated microbiota samples are indicated using dots, triangles, or squares with different shapes representing different patterns of microbiota composition that these samples initially contain. In the expected scenario, the simulated samples stay in the region of their original enterotypes during a long period of time ranging from 5 to 10 years (**A**–**C**). In the observed scenario, the microbiota composition of the simulated samples changes a lot over the time, so that most of them do not stay in the region of enterotypes where they originally belong (**D**–**F**).

After the concept of enterotype was initially proposed [8], the enterotypes of subjects are considered to be stable during a long time, with their microbiota composition varying but within limits. Figure 2A shows that three cohorts of subjects contain three different enterotypes at the beginning. As time evolves, their gut microbiota composition changes, but the extent of this change is not enough to switch the enterotypes. These samples still belong to the groups of their original enterotypes in PCoA, even after many years (Figure 2B and C).

However, subsequent short-term and long-term studies have revealed that such stability is subject to environmental changes, and is not strong in the long run [11], [15], [16], [26]. Dan Knights et al. [16] projected a dense time series of daily gut microbiome samples from a single individual in a year [56] onto the published putative enterotype clusters [8]. Based on the trajectories of microbiome profile from these consecutive daily samples, they have found a switch from one putative enterotype to another over the course of several days [16]. Although validation in a large cohort is warranted, their investigations demonstrate that a certain number of healthy subjects might switch their enterotypes over time, suggesting that the enterotype might not be always stable [16]. As described in Figure 2D and E, as time evolves, a number of subjects change their enterotypes. Two trajectories of switches between enterotypes are shown as examples. The gut sample of a subject represented by the yellow square switches from the original *Ruminococcus* enterotype to the *Bacteroides* enterotype, whereas gut sample of another subject indicated by the yellow triangle switches from the *Bacteroides* enterotype to the *Prevotella* enterotypes. After a certain period of time, enterotypes of these subjects changed completely. As a result, most gut samples of these subjects do not belong to the group that they originally come from (Figure 2F). Therefore, it seems inappropriate to classify people merely according to their enterotypes, due to the instability of enterotypes. Monitoring of enterotype variation of individuals at frequent time intervals is required for further investigations on enterotypes.

### Enterotype might not serve as the biomarker

**Figure 3:**
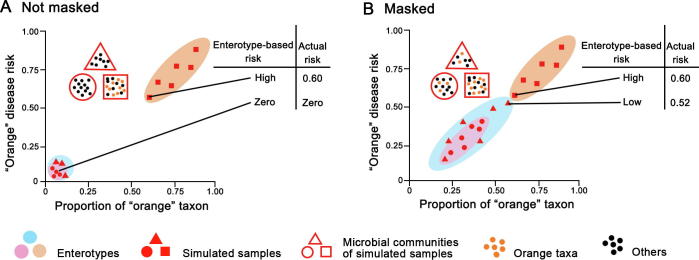
**Enterotype might not be a good biomarker** Simulated gut microbiota samples are plotted as triangles, circles and squares based on their enterotypes. The microbiota composition within these samples determines their enterotypes (*e.g.*, “triangle” samples of “blue” enterotype, “circle” samples of “pink” enterotype, and “square” samples of “orange” enterotype, respectively). These samples disperse on the plots according to the proportion of “orange” taxon (horizontal axis) in their microbiota composition. The “orange” disease risk (vertical axis) is directly associated with the abundance of “orange” taxon. **A.** Under the condition that “orange” taxon is present only in the “square” microbiota, the enterotype-based risk is consistent with actual risk based on the taxon. **B.** Once the “orange” taxon is present in all microbiota samples, the enterotype-based risk might be misleading, which masks the actual risk.

Given enterotypes are defined to indicate clusters composed of gut microbial communities that share similar bacteria composition [8], enterotypes can be used to collapse the highlymultidimensional human microbiome variation into just a few categories. It seems a good idea to use enterotypes as biomarkers that correlate gut microbiome with phenotypes such as diseases. For instance, assuming that the occurrence of certain diseases is observed to significantly correlate with an enterotype, patients then can be grouped according to the enterotypes. Subsequently, a personalized enterotype-based diagnostics and therapeutics would be readily pursued for them. However, enterotypes might not have enough resolution for specific disease-related taxa [16]. In Figure 3, taxon related to a particular disease is depicted in the same color as the risk for this disease. It is shown that the abundance of an “orange” disease-related taxon is correlated directly with the risk of the “orange” disease. In addition, the gut sample that contains dominant “orange” taxon is classified as “orange” enterotype, while the other samples are classified as “pink” or “blue” enterotypes. When determining the disease risk based on taxon, the proportion of “orange” taxon would directly reflect the actual risk of “orange” disease. When determining the disease risk based on enterotypes, the “orange” enterotype would signify high risk and other enterotypes would be the sign as low risk. As shown in Figure 3A, if the “orange” taxon is only present in “orange” enterotype, the enterotype-based risk of the “orange” disease would be consistent with the actual risk. However, as shown in Figure 3B, if the “orange” taxon is present in all enterotypes, enterotype-based risk of the “orange” disease would be likely misleading. Such misleading observation could be explained by the attributes of enterotypes, that enterotypes are not sharply delimited but rather exist as a “broad region”, with even unclear boundary between enterotypes. Hence, if a sample of “blue” enterotype contains a high abundance of “orange” taxon, while this abundance is not high enough to designate this sample as “orange” enterotype, the enterotype-based risk would be low, although the actual risk is high (Figure 3B). Therefore, using enterotypes as the biomarker might mask the real disease risk.

Despite their significant correlation with some diseases, enterotypes might not be appropriate for predicting disease risk due to the masking effects [16]. More investigations are needed to test the feasibility of using enterotypes as the biomarkers.

## Methods and assessments for enterotype analysis

### Methods for enterotype analysis

Metagenomic data or 16S rRNA gene sequencing data are required for enterotype analysis. Accordingly, the built phylogenetic annotation can be used to obtain relative abundances of taxa in gut microbiota at different taxonomic level.

Enterotype analysis was initially conducted at the genus level [8]. First, the abundances of classified genera are used to produce a JSD matrix between samples. Partitioning around medoids (PAM) clustering algorithm is then employed to stratify samples using the distance matrix and the putative number of clusters (designated as ‘k’) as input. To decide the most optimal ‘k’, the Calińksi–Harabasz (CH) index [57] is applied to display how good performance in picking different ‘k’ as the input to PAM. Subsequently, silhouette index (SI) [58] is adopted to assess the statistical validation of their clustering results. Finally, between-class analysis (BCA) and PCoA are performed to visualize the enterotypes.

### Assessments for enterotype analysis

Several factors strongly affect the outcome of enterotype analysis as reported by Koren and colleagues [10]. Firstly, different cluster scoring methods, such as prediction strength (PS) [59], SI [58], and CH [57], could produce inconsistent decisions on the optimal number of clusters when using the same distance matrix. SI and PS provide absolute measures to assess the likelihood of cluster structure emerging from a dataset, whereas CH index provides a relative measure to indicate the optimal number of clusters. Thus, at least one absolute measure (PS for a large-scale of samples and SI for few samples) is recommended to be included in enterotype analysis.

Moreover, using the same cluster scoring method on different distance matrices, such as the JSD, root JSD (rJSD), Bray–Curtis, and weighted/unweighted UniFrac distances, could produce inconsistent results. That means, the detection of enterotype is sensitive to the distance metrics employed. Furthermore, when using different taxonomic levels such as genus level and species level to calculate the distance matrices, inconsistent number of clusters might also be produced in enterotype analysis.

Therefore, various methods should be tested in enterotype analysis to figure out the discrepancies. Due to the lack of a unified standard for methods of enterotype analysis, users should justify their methods of choice.

## Application of the enterotype concept to other organisms

In general, the term “enterotype” refers to our microbiota types within the gut. Interestingly, recently it has been adopted to describe microbiota types across different human body sites [10], even in insects [60] and animals [38].

Several studies have reported the stratification of microbiota in other body sites. For instance, Klatt et al. [61] have successfully stratified 688 HIV-negative women into two clusters, using vaginal microbiota. The first cluster is dominated by *Lactobacillus* and the other one is dominated by non-*Lactobacillus* microbiota. Additionally, two clusters have been identified at oral sites [62], and two clusters have also been recognized in a lung microbiota [63].

As for stratifying microbiota of insects, Li et al. [60] have investigated the gut microbiota of 142 worker bees from 28 species of Chinese bumblebees, and have observed two robust clusters. Most samples (73%) are clustered into a subtype distinguished by abundant *Gilliamella* and *Snodgrassella*, with another subtype containing more *Serratia* and *Hafnia*. Both clusters share *Lactobacillus*.

For animal studies, Moeller et al. [38] have investigated the gut microbiota of 35 chimpanzees from the Gombe Stream National Park. They find that microbiota profiles of these chimpanzees can be stratified into three clusters, with dominant *Faecalibacterium*, *Lachnospiraceae*, and *Bulleidia*, respectively. It is of note that the microbiota clustering is not significantly related to the age, genealogy, or gender of their hosts.

## Conclusion and perspectives

Enterotype has remained a controversial concept as to whether human gut microbiome can be clustered into different types or just fall into a continuous gradient.

Owing to the extreme complexity of highly dimensional microbiota in human guts, it is really pragmatic for researchers to collapse them into a few categories. Nevertheless, existing studies cannot either substantiate or deny the enterotype concept. With more experiments conducted at a larger space scale to validate and improve the enterotype concept, it would be feasible to realize the categorization of human gut microbiota, using a unified enterotype method. This categorization would help us further understand the correlations between gut microbiota and diseases to facilitate precision medicine based on gut microbiota. A recent work has already made a concrete step toward this goal [54]. According to a classification procedure of enterotypes proposed by Costea et al. [54], if new gut samples cannot overlap with the enterotypes in the provided reference set, the enterotypes of these new samples will be computed *de novo*. Otherwise, these samples will be assigned to existing enterotypes of the reference set. This study provides the possibility for a unified standard of enterotypes analysis.

However, a number of technical issues would impact the enterotype analysis. For instance, the input datasets of enterotype analysis are directly influenced by multiple factors including the sample processing, DNA extraction, and the sequencing technology [64]. Moreover, relative abundance, rather than the absolute abundance, is adopted in enterotype analysis due to the technological hurdles in obtaining the absolute abundance, which might not describe real profiles of microbiota. Using more advanced techniques such as flow cytometry, we can now perform enterotype analysis based on the absolute number of taxa [65].

As we have described, the concept of enterotype can be applied not only in human gut microbiota but also in microbiota samples from other human body sites. Thus, we expect in other ecological niches, microbiota might also be stratified into different subtypes designated as “soiltype” (in soil), “marinotype” (in marine), “plantotype” (in plant), *etc*. Collapsing the highly multidimensional microbiota of ecological niches into a few categories might help us to better describe the characteristics of these microbiota, and then deal with environmental issues.

Finally, we have to admit that gut microbiota changes constantly in a dynamic status. Considering this, more studies are supposed to focus on the dynamic nature, using frequent sampling, with integrative comparison of microbiota on time-series or among the changing conditions. This would be a critical step toward a comprehensive understanding of the ecology and evolution of any microbiota.

## Competing interests

The authors have declared no competing interests.
